# Editorial: Risk of COVID-19 transmission to oral healthcare providers and their patients - Volume II

**DOI:** 10.3389/froh.2023.1266336

**Published:** 2023-08-30

**Authors:** Joana Cunha-Cruz, Marilynn L. Rothen

**Affiliations:** ^1^ Department of Clinical and Community Sciences, University of Alabama at Birmingham, Birmingham, AL, United States; ^2^ Department of Oral Health Sciences, University of Washington, Seattle, WA, United States

**Keywords:** COVID-19, SARS—CoV-2, editorial, qualitative study, survey, dental personnel, PPE (personal protection equipment)

**Editorial on the Research Topic** Risk of COVID-19 transmission to oral healthcare providers and their patients - Volume II

Last year, the collection of peer reviewed articles in volume one of this research topic (Rothen and Cunha-Cruz) focused on reducing the risk of COVID-19 transmission through infection control (Otieno et al.) and clinical guidelines (Luo et al.; Pittayapat et al.) and on understanding the impact of fear, uncertainty, and economic hardship on dental care providers (Humphris et al.; Knights et al.). Volume two provides different perspectives on the many lessons learned during and after the global COVID-19 pandemic. The studies come from a variety of countries and use different study designs, but they all provide valuable information about the challenges and experiences of dental professionals during the pandemic.

• “**Paradigm shift in infection control practices in dental clinics in response to COVID-19 among dental professionals in Thailand”** was a cross-sectional study of 1,177 dental professionals in Thailand. The study found that Thai dental professionals have significantly increased their infection control practices in response to the pandemic, with the infection rate among dental professionals in Thailand being lower than the general population.
• “**Utilization of rapid antigen tests for screening SARS-CoV-2 prior to dental treatment”** was a cross-sectional study of 7,618 patients screened for SARS-CoV-2 and a questionnaire study with dental personnel in an academic dental clinic in Thailand. After describing their procedures in Volume 1 (Pittayapat et al.), the authors found, in Volume 2, that rapid antigen tests can be used to screen for SARS-CoV-2 prior to dental treatment and are well received by the dental personnel, but only a small number of patients tested positive, and the appointment time increased.
• “**Longitudinal online diaries with dental practitioners and dental care professionals during the COVID-19 pandemic: A trajectory analysis”** was a longitudinal study of 110 dental trainees and primary dental care staff in the United Kingdom. Expanding the cross-sectional analyses presented in Volume 1 (Humphris et al.; Knights et al.), the study found that dental professionals experienced a great deal of uncertainty and anxiety during the early stages of the pandemic, but these feelings decreased over time as dental professionals adapted to the new normal. They also found that dental professionals were concerned about the impact of the pandemic on their patients' oral health, and they were also concerned about the financial implications of the pandemic.
• “**Dentists’ lived experience of providing dental care during the COVID-19 pandemic: A qualitative study in Mashhad, Iran”** was a qualitative study of 16 dentists in Iran. The study found that dentists experienced several challenges during the pandemic, including increased workload, changes in work procedures, and concerns about the safety of their patients and themselves. However, the study also found that dentists were able to adapt to the new challenges and continue to provide high-quality dental care.

Taken together, the studies in this second volume suggest that dental professionals have taken significant steps to adapt to new guidelines to increase infection control measures and protect their patients (Amnuaiphanit et al.) including the use of SARS-CoV-2 screenings prior to treatment (Pittayapat et al.). Dental professionals continued to experience a great deal of uncertainty and anxiety, but these feelings decreased over time as they adapted to the new normal (Beaton et al.; Amnuaiphanit et al.; Hoseinzadeh et al*.*).

The findings of these studies highlight the importance of providing support to dental professionals during this challenging time. This support can come in a variety of forms, including financial assistance, mental health counseling, and training on new infection control measures. In addition to the support, it is also important to recognize the resilience of dental professionals. Despite the challenges they have faced, dental professionals have shown a great deal of adaptability and determination. They have continued to provide essential dental care, and they have also played a role in educating the public about COVID-19. The COVID-19 pandemic has been a difficult time for everyone, and the articles demonstrated the unique set of challenges faced by dental professionals.

